# Clinical updates on tyrosine kinase inhibitors in HER2-positive breast cancer

**DOI:** 10.3389/fphar.2022.1089066

**Published:** 2022-12-12

**Authors:** Desh Deepak Singh, Hae-Jeung Lee, Dharmendra Kumar Yadav

**Affiliations:** ^1^ Amity Institute of Biotechnology, Amity University Rajasthan, Jaipur, India; ^2^ Department of Food and Nutrition, College of Bionano Technology, Gachon University, Seongnam-si, Gyeonggi-do, South Korea; ^3^ Department of Pharmacy, Gachon Institute of Pharmaceutical Science, College of Pharmacy, Gachon University, Incheon, South Korea

**Keywords:** breast cancer, HER2-positive, tyrosine kinase inhibitors, novel combinations, clinical practice

## Abstract

Breast cancer (BC) is caused by epigenetic modifications and genetic heterogeneity and exhibits various histological feature. HER2+ (Human epidermal growth factor receptor 2) is a more aggressive type of breast cancer, diagnosis and prognosis are difficult for HER2+ BC. Anti-HER2+ inhibitors have been effectively used for patient treatment. High mortality rate is reported in HER2+ BC, due to availability of limited therapeutic options. Despite advances in systemic medications to treat metastatic breast cancer (MBC), HER2-positive MBC is still challenging for patients and treating clinicians. The clinical characteristics of the disease have changed after treatment with HER2-targeted therapy. Various types of Tyrosine kinase inhibitors (TKIs) have been developed to treat patients with HER2+ BC including afatinib, lapatinib, neratinib, tucatinib, and pyrotinib, have been developed as HER2-targeted therapies. The antibody-drug conjugates adotrastuzumab, emtansine, famtrastuzumab, and deruxtecan, as well as the anti-HER2 monoclonal antibody pertuzumab are used in both early-stage and metastatic situations, either alone or in conjunction with chemotherapy and other HER2-targeting therapies. The emergence of drug resistance in anti-HER2 therapies has been observed. To overcome drug resistance and limited efficacy in current treatment options, nano formulations can be used in patients with HER2+ BC treatment. Anti-HER2 ligands can be used in various nano formulations to target HER2 receptors. Here we will discuss, targeted TKIs in patients with HER2+ BC, clinical studies of HER2+ targeted TKIs, mechanisms of resistance to HER2-directed therapies with new implications of TKIs in HER2+ MBC (metastatic breast cancer) and anti-HER2 ligand in various nano formulations to target HER2 receptors.

## 1 Introduction

**FIGURE 1:**
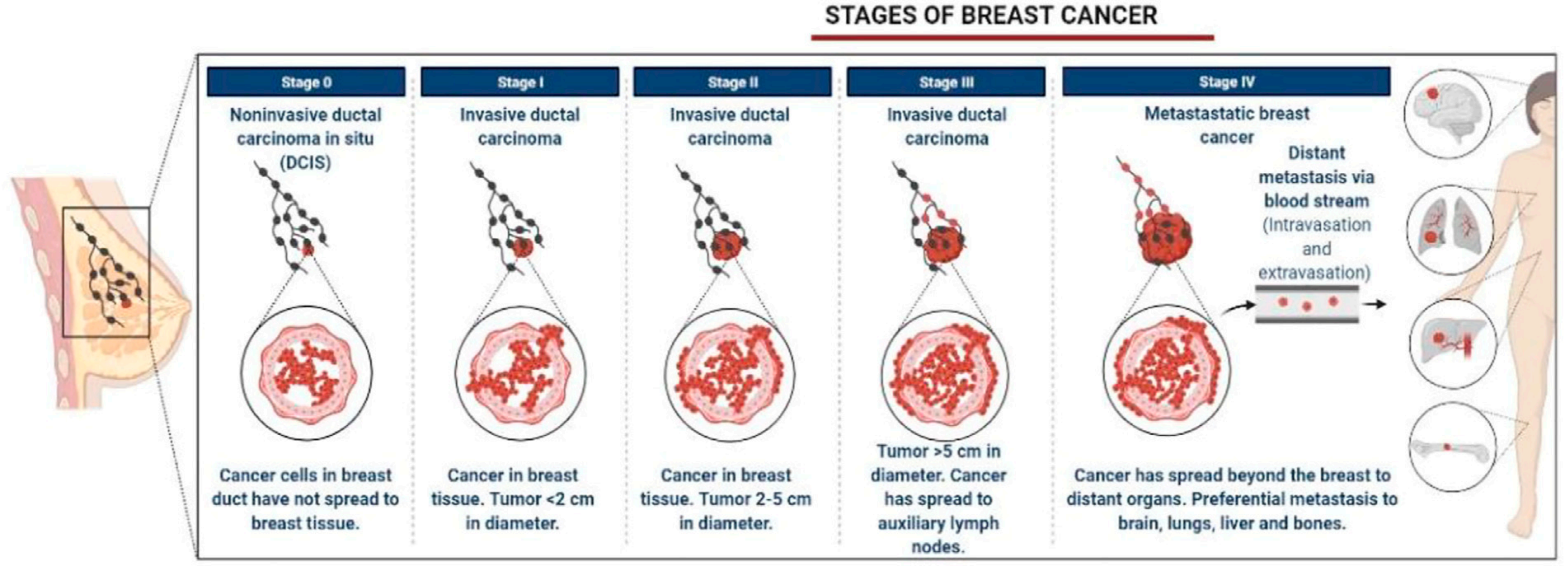
Various stages of breast cancer.

**FIGURE 2:**
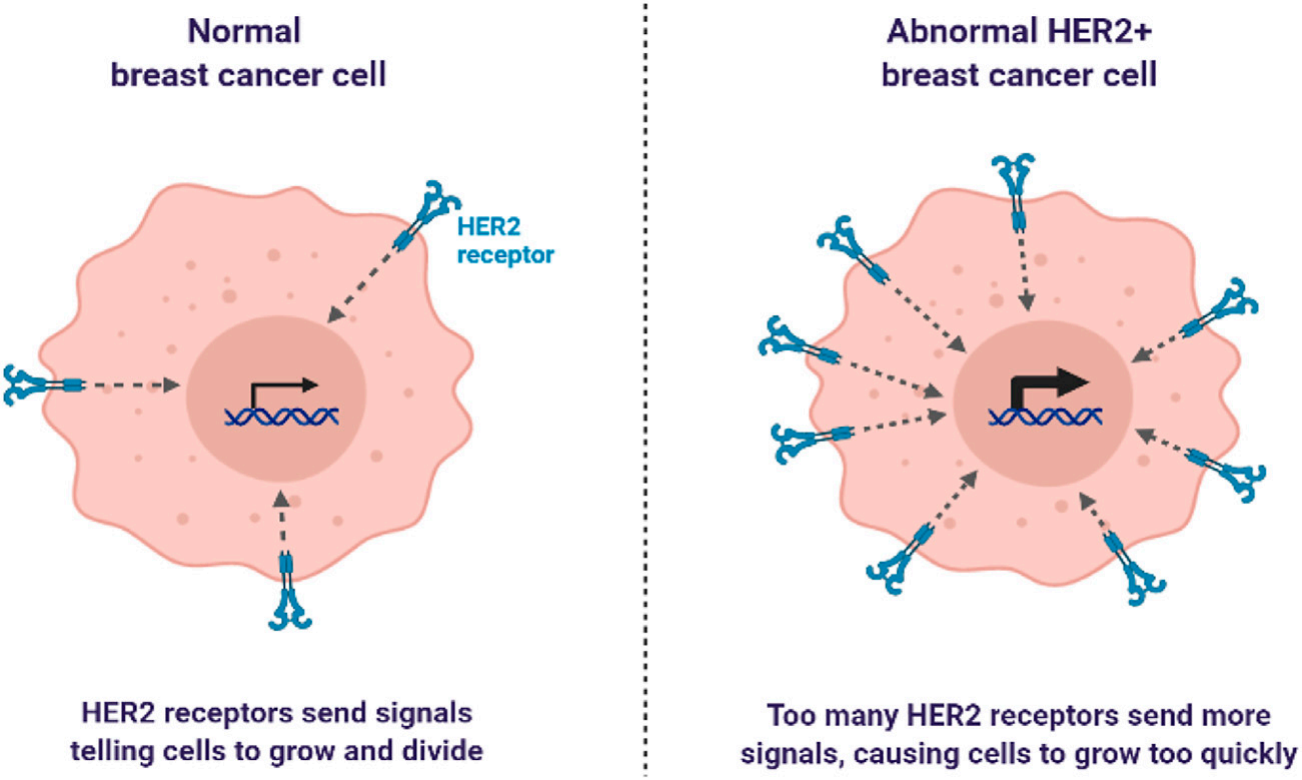
Representation of normal breast cancer cell and abnormal breast cancer cell.

**FIGURE 3:**
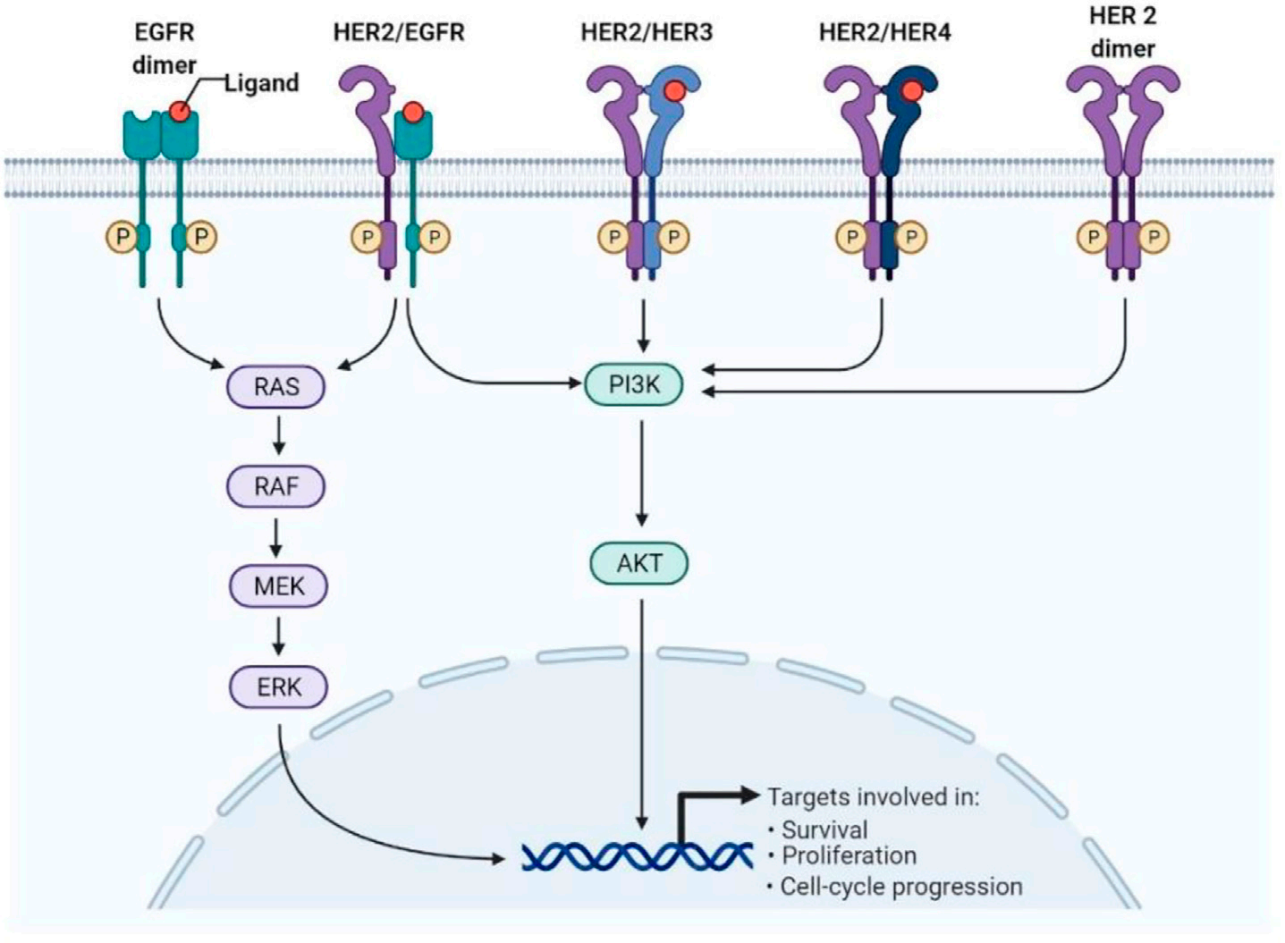
Tyrosine kinase inhibitors against HER2+ BC.

**FIGURE 4:**
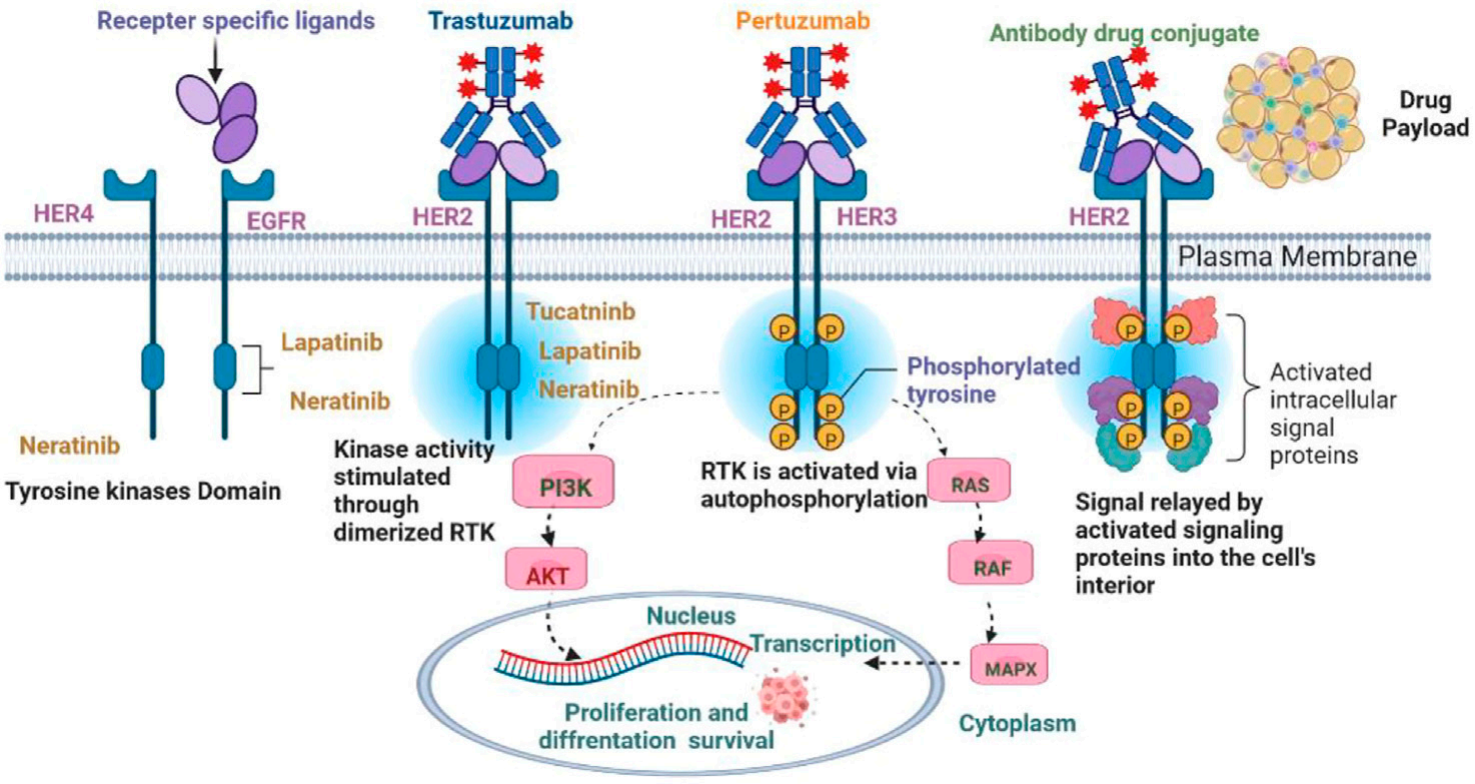
Signaling pathways in HER2+ breast cancer.

BC is characterized by the growth and development of cells with distinctive genetic and clinical characteristics (Harbeck and Gnant, 2017). BC is developed in four stages with different clinical characteristics, as shown in Figure 1 (McDonald et al., 2016). HER2+ BC expresses the HER2 protein receptor (Loibl and Gianni, 2017). Figure 2 illustrates that many HER2 receptors deliver signals, as compared to normal HER2 receptors, that provide signals guiding cells to expand and divide, leading abnormal HER2+ BC cells to develop very quickly (Waks and Winer, 2019). Brain metastases (BM) have been observed in 30% of patients with HER2+ BC. The four types of epidermal growth factor receptors are HER1, HER2, HER3, and HER4, as shown in Figure 3 (McDonald et al., 2016). The extracellular ligand-binding and intracellular kinase domains of HER family receptors are homologous, they are expressed in a particular cell type, and they are phosphorylated *via* heterodimerization events started by specific ligands (Seshacharyulu et al., 2012). These receptors are distinct from one another in their intracellular c-terminal signalling (McDonald et al., 2016; Loibl and Gianni, 2017). The most effective activator of the PI3K/AKT (Protein Kinase B, PKB) signaling cascade is the HER2 heterodimer, which binds to the PI3K (Phosphoinositide 3-Kinases) p85 subunit. (Wolff et al., 2018). HER2+ patients are difficult to treat due to their poor prognosis. Effective treatments are developed by reviewing and elucidating mechanisms of resistance and identifying new approaches by blocking the signal transduction mechanism by using HER2 and related pathways (Sung et al., 2018). However, after the development of HER2-targeted therapy, the survival rate of HER2-positive breast cancer patients has considerably improved (Waks and Winer, 2019). As a result, HER2-targeted therapy has changed the clinical symptoms of the illness. Effective treatments are established by assessing and understanding the causes of resistance and discovering a novel strategy for blocking signal transduction mechanisms using HER2 and related pathways (Vernieri et al., 2019). Several HER2 targeted treatments, including TKIs, have been developed in recent years (Waks and Winer, 2019). TKIs are used to inhibit cancer cell growth by reducing TK phosphorylation and competing with RTKs’ (receptor tyrosine kinases’) ability to take up and use oxygen at the ATP (adenosine triphosphate) binding site (El Guerrab et al., 2016). Targeted anti-EGFR (Epidermal Growth Factor Receptor) therapies are used as TKIs, such as osimertinib, erlotinib, neratinib, tucatinib, gefitinib, canertinib, afatinib, lapatinib, and pyrotinib, as well as monoclonal antibodies (Figures 3, 4). Various clinical trials are being investigated for their clinical potential in Table 1 (Schlam and Swain, 2021). Following a clinical trial, the FDA (Food and Drug Administration) has approved tratuzumab for the management of advanced HER 2+ BC (Tan et al., 2021). The combined therapy of trastuzumab, pertuzumab, and taxane is now the gold standard for treating MBC with HER2, regardless of whether the patient has HER2+ or HER2-negative MBC (Jagosky and Tan, 2021). There is some evidence that TKIs are more easily able to traverse the blood-brain barrier than larger molecules like monoclonal antibodies or antibody-drug conjugates (Tan et al., 2021). The effectiveness of various HER2-targeted TKIs has been studied, as indicated in Tables 1, 2. T-DM1 (ado-trastuzumab emtansine), which was previously used to treat patients with HER2-positive metastatic breast cancer treated with trastuzumab and taxane chemotherapy, was found to be safe and effective in the EMILIA (Emtansine Versus Capecitabine + Lapatinib in Participants with HER2-positive Locally Advanced Cancer) trial (Table 1). On 22 February 2013, the FDA authorised the first antibody conjugate (Verma et al., 2012). There has been no well-established course of treatment since the onset of TDM1 progression. However, anti-HER2 therapy should be continued. Trastuzumab with other chemotherapeutic medications, anti-HER2 antibody conjugates with trastuzumab diluxtecan, or small molecule HER2-TKI with other treatments are among the treatment possibilities for these situations (Figure 2) (Wang and Xu, 2019). In HER2+ MBC, the effectiveness of several TKIs was demonstrated. These TKIs, on the other hand, target HER family proteins in diverse ways, necessitating the development of tailored treatment regimens. Here, we will emphasize Receptor Tyrosine Kinases (RTK), TKIs Therapy for HER2+ MBC, HER2-targeted TKIs are being studied in key phase 3 clinical trials in BC, TKIs and Brain Metastasis, Mechanisms of Resistance to HER2-Directed Therapies, clinical studies of HER2+ targeted TKIs, with new implications of TKIs in HER2+ MBC and nanomedicine to treat HER2+ BC.

**TABLE 1:** HER2-targeted TKIs are being studied in key phase 3 clinical trials in breast cancer.

Trial information	Drug combinations in tested papulations	Sample size	Population	Findings	References
Lapatinib					
ALTTO	Comparing trastuzumab plus lapatinib (TL) to trastuzumab (T) alone against trastuzumab plus lapatinib (TL)	8,381	HER2+, >1 cm localized BC	There was no difference in disease-free survival between T alone and T plus lapatinib (*p* = 0.61)	Li et al. (2020)
CEREBRAL	Lapatinib plus capecitabine (LC) against trastuzumab plus capecitabine (TC)	540	Metastatic BC with HER2 positivity and no prior history of brain metastases	BM and other locations did not differ in the first site of relapse (*p* = 0.360). Both the OS (overall survival) and PFS are greater for the TC arm (progression-free survival)	Khan et al. (2020)
NeoALTTO	Trastuzumab, Lapatinib (L), and Lapatinib (TL)	455	HER2+ females with localized BC more than 2 cm	TL had a pathologic complete response (PCR) in 51.3% of cases compared to T’s 29.5% (*p* = 0.0001)	Baselga et al. (2012)
	Trastuzumab is also used in post-neoadjuvant (taxane) and neoadjuvant (plus taxane) situations (T)				
GeparQuinto GBG 44	As a neoadjuvant therapy, EC (Epirubicin, Cyclophosphamide), docetaxel, and trastuzumab (T) against lapatinib were administered (L)	620	Lymph nodes, HER2, and BC women	30.3% in T and 22.7% in L for PCR (0.04)	Schlam and Swain, (2021)
CALBG 40,601	In the neo-adjuvant situation, paclitaxel plus lapatinib plus trastuzumab (THL) vs. paclitaxel plus lapatinib (TL) vs. paclitaxel plus trstuzumab	305	Left ventricular ejection fraction >50%, HER2+, >1 cm localized BC	56% TPL and 46% TP, PCR (*p* = 0.13). After 7 years of follow-up, the triplet showed an increase in recurrence-free survival and overall survival	Golshan et al. (2016)
NSABP B-41	Neoadjuvant doxorubicin, cyclophosphamide, weekly taxanes, followed by lapatinib (TL) with trastuzumab, versus trastuzumab with alone	130	Positive for HER2, localized BC > 2 cm, and left ventricular ejection fraction >50%	PCR 52.5% (*p* = 0.95) of T, 53.3% of L, and 62% of TL	Swain et al. (2020)
TEACH	Adjuvant chemotherapy followed by lapatinib and a placebo	3,147	Stage I-IIIc BC, not previously treated with trastuzumab	17% placebo vs. 13% lapatinib for disease-free survival (*p* = 0.53; no difference in OS (overall survival) or time to first recurrence)	Lee et al. (2018)
Metastatic					
COMPLETE	Contrast lapatinib plus paclitaxel with trauzumab plus capecitabine	537	HER2+, metastatic BC	PFS for the trastuzumab group was 13.6 months compared to 9 months for the lapatinib arm (HR 1.48, *p* = 0.001)	Mehta et al. (2019)
Lapatinib plus letrozole	Lapatinib plus letrozole (LL) against placebo plus letrozole (LP)	219	HR+, HER2+, or metastatic BC	For HER2+ patients, the progression-free survival was 8.2 months in LL and 3 months in LP.	Park et al. (2016)
Lapatinib plus capecitabine	Capecitabine vs. Lapatinib (CL) plus Capecitabine (C)	324	A patient with metastatic breast cancer who tested positive for HER2 and responded to chemotherapy and trastuzumab	Time to progression was 8.4 months in LC and 4.4 months in C	Cetin et al. (2014)
Neratinib					
NALA	In contrast to lapatinib with capecitabine (LC), neratinib plus capecitabine (NC)	621	Patients with metastatic BC who were HER2+ received two courses of HER2-directed therapy	PFS was greater with NC (*p* = 0.0059). OS was identical between the two groups; the response lasted 8.5 months in NC and 5.6 months in LC (*p* = 0.0004). (*p* = 0.043) NC received fewer CNS (Central Nervous System) therapies than LC.	Dai et al. (2021)
NEfERT-T	Neratinib and paclitaxel (NP) against trastuzumab and paclitaxel (TP)	479b	Previously untreated HER2+ metastatic BC	The median PFS for NP was 12.9 months, whereas the median PFS for TP was 12.9 months. The NP group had a lower incidence of central nervous system metastases (relative risk 0.48, *p* = 0.002)	Awada et al. (2016)
ExteNET	Neratinib (N) was utilized as adjuvant medication for a year instead of a placebo (P)	2,840	HER2+, stage I–III BC patients who finished their course of treatment, which included trastuzumab for a year	DFS was lower in the N group (*p* = 0.0091). Less than a year after starting trastuzumab, patients who began treatment had an absolute advantage of 7.4% in if and 9.1% in OS after eight years	Martin et al. (2017)
Tucatinib					
Metastatic HER2CLIMB	Contrasts between placebo plus trastuzumab + capecitabine and TTC (tucatinib plus trastuzumab plus capecitabine) (PTC)	612	Two lines of HER2-directed treatment were given to HER2+ metastatic BC patients	In comparison to PTC, TTC had a 33.1% PFS after one year (*p* = 0.001)	Lin et al. (2020)
				Compared to 5.6 months, TTC 7.8 months had a better PFS.	
				OS in TTC was 44.9% and in PTC it was 26.6% (*p* = 0.005)	

**TABLE 2:** Clinical trials on HER-2-targeted tyrosine kinase inhibitors for BC.

Clinical trial identification number	Phase	Study type	No of participant	Interventions	End point analysis
NCT01042379	Phase -II	Randomized	4,000	Tucatinib, trastuzumab, pertuzumab, paclitaxel, doxorubicin, and cyclophosphamide as neoadjuvant therapy	PCR (pathologic complete response) RBC, OS (overall sruvuival), safety, and relapse-free survival
NCT03101748	Phase -II	Non-randomized	43 participants	Neratinib and paclitaxel with or without pertuzumab and trastuzumab before combination chemotherapy in treating patients with MBC	PCR maximum tolerated dose of neratinib, PFS (progression-free survival), safety
NCT04457596	Phase -III	Randomized	1,013 participants	Adjuvant tucatinib plus emtansine and ado-trastuzumab against ado-trastuzumab emtansine for patients with post-neoadjuvant illness that has relapsed	Aggressive DFS (disease free survival) OS metastasizes in distant DFS, CNS
NCT03085368	Phase -III	Randomized	482 participants	Lapatinib with paclitaxel vs herceptin and paclitaxel with sequential and synchronous anthracycline for HER-2 positive breast cancer patients	DFS, OS
NCT01670877	Phase -II	Non-randomized	56 participants	Neratinib study in metastatic HER2 “non-amplified” but HER2 mutant breast cancer: neratinib alone and in combination with fulvestrant	ORR (overall response rate) PFS, safety
NCT03975647	Phase -II	Randomized	565 participants	Ado-trastuzumab emtansine (T-DM1) with tucatinib or placebo for patients with unresectable locally advanced or metastatic HER2+ breast cancer (HER2CLIMB-02)	PFS OS, PFS per RECIST (Response evaluation criteria in solid tumours), ORR, DOR (duration of response), clinical benefit, adverse events
NCT04539938	Phase -II	Open label	70 participants	Combining tucatinib with trastuzumab deruxtecan in advanced or metastatic patients with locally advanced HER2+ breast cancer after preliminary treatment	ORR DOR, PFS, disease control rate, OS, adverse events
NCT03054363	Phase -II	Non-randomized, open-label single arm	42 participants	Tucatinib, palbociclib, and letrozole combination study to assess safety and efficacy in patients with estrogen receptor positive and HER2-positive MBC	Tolerability\sPFS
NCT01494662	Phase -II	Non-randomized	140 participants	HER2-+ BC patients with MBC may benefit from the use of the drugs HKI-272 (Neratinib), neratinib with capecitabine, and ado-trastuzumab emtansine	Clinical results: ORR PFS, OS, CNS response, location of progression, safety, and tolerability
NCT04334330	Phase -II	A multi-centric, prospective	34 participants	Effects of trastuzumab, pyrotinib, and fulvestrant on patients with brain metastases from ER/PR positive, HER-2 positive BC.	Safety, OS, PFS, ORR, progression time, and radiation treatment time
NCT03501979	Phase -II	Non-randomized	30 participants	Leptomeningeal metastases in HER2 + BC: A tucatinib, trastuzumab, and capecitabine combination	OS safety, PFS, and CNS ORR, quality of life, clinical benefit, symptom severity
NCT04512261	Phase -II	Single arm, open label	Recruiting	Patients with HER2-positive MBC received tucatinib in combination with pembrolizumab and trastuzumab	ORR, PFS, OS, toxicity profile

## 2 Receptor tyrosine kinases

There are 518 kinase genes in humans, and half of them are known as receptor tyrosine kinases (McDonell et al., 2015). The functions of the protein tyrosine kinases (PTK) include cell differentiation, metabolism, growth, response to stimuli, and adhesion (Wang et al., 2016; Kim et al., 2017). A molecular mechanism can lead to a dysregulated signal cascade mechanism, mainly resulting in malignancy and other pathologies. TKIs compete with ATP for the ATP binding site of PTK and completely block PTK-mediated signalling pathways, inhibiting cancer cell proliferation (Alexander et al., 2017; Yang et al., 2022). TKIs are more efficient than conventional treatments, dimers activate the HER2 extracellular domain, causing TK residues in the cytoplasmic domain to be phosphorylated (Alexander et al., 2017; Alexander et al., 2017). These residues serve as docking sites for proteins that activate the phosphatidylinositol triphosphate kinase (PI3K) and mitogen-activated protein kinase (MAPK) signalling pathways, promoting cell cycle progression and proliferation (Arcaro and Guerreiro, 2007). The two main pathways, RAS (Reticular Activating System)/MAPK and PI3K/AKT, are actively involved in angiogenesis, proliferation, and regulating various cell signalling mechanisms (Arcaro and Guerreiro, 2007; Alexander et al., 2017). The TKI is fully capable of reversing these mechanisms, modulating the immune suppressive tumour environment, and enhancing the anti-tumor property (Kwilas, et al., 2015; Wang et al., 2016). According to the findings of a clinical trial (NCT0301333), TKIs and antibiotics improve the therapeutic response. Glioblastoma (GBM) cell lines have produced various outcomes in a different study (Schlam and Swain 2021). Although different TKIs have a comparable set of receptor-specific targets, non-derepressible 2 (GCN2), an activated integrated stress response (ISR), is a factor that can be regulated by TKIs and results in cell death (Wang et al., 2016; Tian et al., 2021). At the same time, TKIs have shown different roles in pharmacokinetics and target different kinases. Some TKIs have shown more potential with multiple targets as shown in Figures 3, 4. Sorafenib targets vascular endothelial growth factor and PDGFR (platelet-derived growth factors), which inhibit tumorigenesis as shown in Table 3 (Klempner et al., 2013; Wang et al., 2016). Osimertinib is a third generation TKI that targets EGFR (Wang et al., 2016), Imatinib is more specific for BCR-ABL mutations, more than 75 types of TKIs are used to treat various types of cancer, and most of them are under clinical trial in various phases shown in Table 2 (Bhullar et al., 2018). TKIs have only mild non-specific toxicity, and due to safety concerns, they can be used in combination with other therapeutic modalities such radiation, chemotherapy, and immune-based therapies. TKIs have had positive outcomes in the management and care of patients (Tables 1–4) (Bhullar et al., 2018; Canet et al., 2021). TKs are implicated in several malignancies and are classified into two types, such as receptor protein kinases (RTKs) and non-receptor protein kinases (NRTKs), When ligands bind to activated RTKs, they act as cell-surface signal transducers (Siveen et al., 2018). Transmembrane proteins have been identified, that have ligand-binding domains outside of the cell membrane (Trenker and Jura, 2020). A transmembrane domain (TMD) is a protein domain that spans a membrane, although some TMDs, like those in porins, can adopt a different conformation. Most TMDs typically adopt an alpha helix topological configuration (Fink et al., 2012). The transmembrane domain serves as an anchor and is placed close to the protein’s N-terminus. A single transmembrane helix links an external ligand-binding domain to an intracellular domain that contains the peri-membrane regulatory region tyrosine, which is a common feature among RTKs (Hsu and Hung, 2016; Christensen et al., 2017). The involvement of multiple RTKs in the development and spread of neoplasia makes them potential anticancer treatment targets (Hsu and Hung, 2016; Pottier et al., 2020). RTK inhibition therapy is used to treat cancer, although acquired and adaptive resistance persists, TKIs give remission for most cancer patients. If there is no plan, there will be resistance to concentrated therapy (Ahearn et al., 2018; Yamaoka et al., 2018). The inability to manage dormant cancer cells makes the diagnosis, treatment with targeted immunotherapies, and chemotherapy more challenging (Pottier et al., 2020). The main trigger of phosphorylation is included epigenetic controls, the tumour microenvironment, and the modification of cytogenetic and genetic mutations (Hsu and Hung, 2016; Ardito et al., 2017). Following ATP hydrolysis, the protein accepts a phosphate group, and because of the enzymatic activity of kinase and the activity of phosphatase, the process of phosphorylation is reversible (Hunter, 2012). The phospho-binding proteins bind with the phosphate group of a phosphoprotein, dephosphorylation and phosphorylation are both changes at molecular level. The trans autophosphorylation is caused by autoinhibitory sites due to RTK ligand-induced dimerization, develop PTM (post-translational modification), and leads to activation of the oncogenic pathway, as shown in Figure 3 (Ahearn et al., 2018; Yamaoka et al., 2018).

**TABLE 3:** TKIs are used in the treatment of HER2+ metastatic breast cancer.

TKI	Maximum dose concentration (MDC)	HER2 targets (IC_50_)	Mechanism of binding	References
Lapatinib	1,250/1,500 mg	+++ 9 nM	Reversible	Khan et al. (2020)
Neratinib	240 mg	+ 59 nM	Irreversible	Dai et al. (2021)
Tucatinib	600 mg	+++ 8 nM	Reversible	Lin et al. (2020)
Pyrotinib	400 mg	+++ 38 nM	Irreversible	Wang et al. (2022)
Afatinib	40 mg	++ 14 nM	Irreversible	Wind et al. (2017)

Activity scale: IC50 <10 nM = +++ (highly active); 10 nM ≤ IC50 < 100 nM = ++ (moderately active); IC50 ≥ 100 nM = + (low active).

**TABLE 4:** List of monoclonal antibodies approved by the FDA.

S.N.	Types of monoclonal antibodies	Name of antibodies	Name of antibodies antigen	Approved against types of cancer
1	Humanized IgG1	Atezolizumab	PD-L1	Triple-negative breast cancer
2	Humanized IgG1	Trastuzumab	HER2	Breast cancer
3	Humanized IgG1	Pertuzumab	HER2	Breast cancer
4	Humanized ADC	Trastuzumab emtansine	HER2	Breast cancer
5	Humanized ADC	Trastuzumab deruxtecan	HER2	Breast cancer
6	Humanized ADC	Sacituzumab govitecan	TROP2	Triple negative breast cancer

**FIGURE 5:**
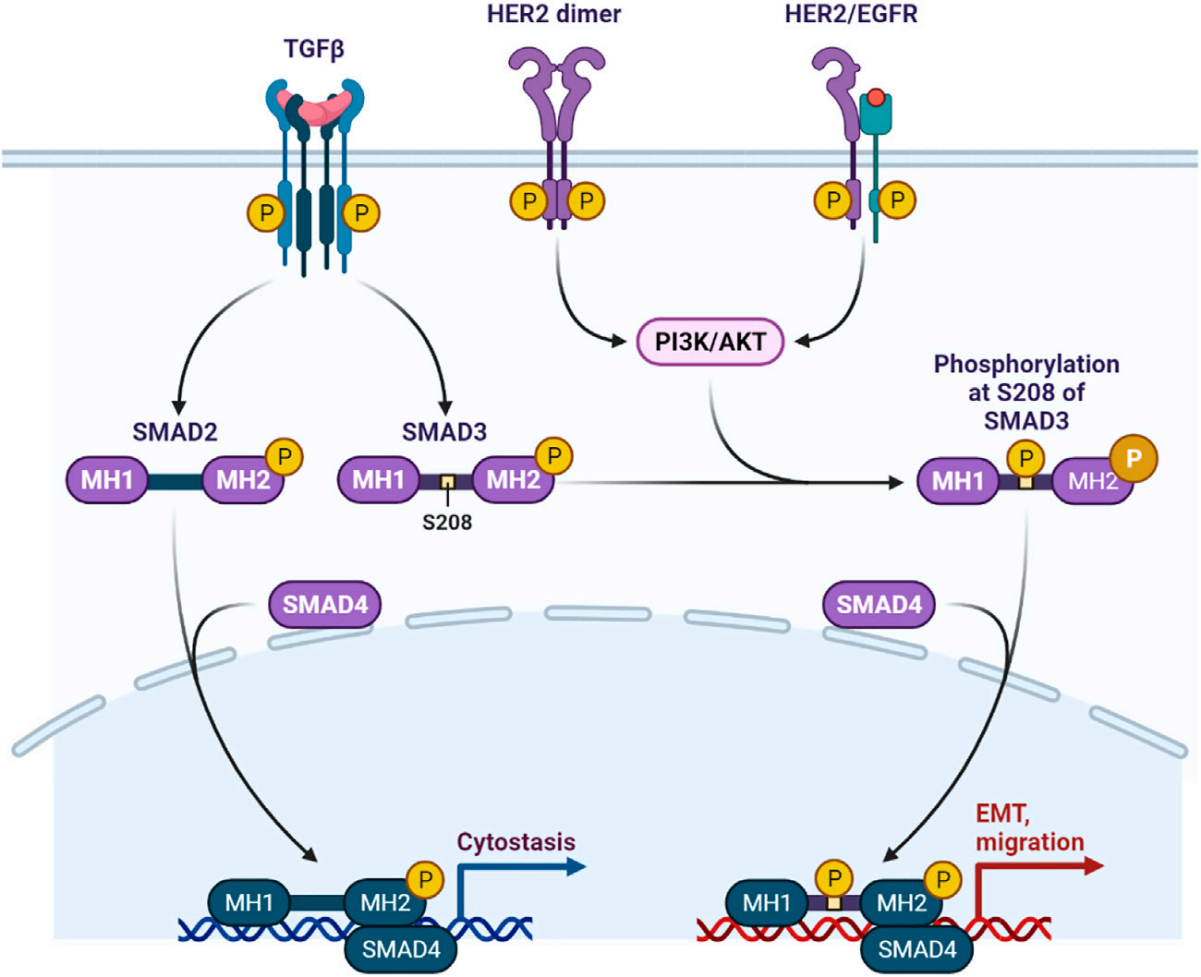
HER2/EGFR signaling pathway in breast cancer.

The SH2 (Src homology region 2) and PTB (phosphotyrosine-binding) domains of proteins attach to the phosphorylated side chains of tyrosine (Jin et al., 2015). Tyrosine phosphorylation is a crucial factor in the eradication of auto-inhibition of auto-phosphorylation (Gal-Ben-Ari et al., 2019). The enormous assortment of RTK activation pathways is due to several RTK domains and a variety of ligand-binding modalities (Hsu and Hung, 2016; Du and Lovly, 2018). RTK activation relies heavily on the phosphorylation of tyrosine residues in the cytoplasmic domain, as shown in Figures 4, 5 (Hsu and Hung, 2016). From immunoglobulin-like (Ig-like) folds and other folds to RTKs, their extracellular domains differ in a wide range of ways.

## 3 Tyrosine kinase inhibitor therapy for HER2+ MBC

Treatment of breast cancer with trastuzumab, the first HER2-targeted drug, began in the late 1990s. HER2-based therapeutic options have greatly shifted for the clinical management of patients with HER2+ (Bredin et al., 2020). Trastuzumab, the first HER2-targeted medication, was first used to treat breast cancer in the late 1990s. Options for HER2-based therapy for the clinical care of patients with HER2+ have significantly changed. For the treatment of patients with HER2+ MBC, the TKIs lapatinib, neratinib, and tucatinib have demonstrated efficacy and acquired regulatory approval as shown in Tables 1, 2 (Sung et al., 2018). The therapeutic efficacy of pyrotinib and afatinib is still being studied, the antibody-drug conjugates adotrastuzumab emtansine and famtrastuzumab deruxtecan, as well as the anti-HER2 monoclonal antibody pertuzumab, have all received approval (Wang et al., 2022). Additionally, a HER2-targeting antibody, Margetuximab, was recently licenced for use in the treatment of HER2-positive breast cancer. Patients with early or advanced HER2+ breast cancer may benefit from low molecular weight TKIs as well (Pernas and Tolaney, 2019). For the treatment of lung cancer with an EGFR, HER2, or HER4 mutation, afatinib has been authorised, Afatinib’s early trials yielded promising outcomes. Phase 3 studies, however, were unable to demonstrate any advantage for those with advanced breast cancer (Wind et al., 2017). TKIs are efficacious as monotherapy or in combination with chemotherapy and other HER2-targeting therapies, as indicated in Tables 1–4 (Schlam and Swain, 2021). A HER2-targeted TKI with or without trastuzumab may be advantageous for patients who are responding well to trastuzumab, according to studies (Tan et al., 2021). The clinical trials and therapies for HER2+ MBC are listed in Table 1. Here, we discuss the clinical application, efficacy data, and safety profiles of the TKIs in patients with HER2+ MBC*.*

### 3.1 Lapatinib

Lapatinib is the second anti-HER2 drug after trastuzumab (Baselga et al., 2021) used for the patients with advanced HER2+ or MBC who have already received anthracycline, taxane, or trastuzumab therapy may combine lapatinib and capecitabine therapy (Khan et al., 2020; Baselga et al., 2021). Lapatinib and letrozole have been approved for use in women with postmenopausal hormone receptor positive (PHER2+) MBC (Khan et al., 2020; Baselga et al., 2021). Additionally, the combination of lapatinib plus trastuzumab is authorised in the EU (European Union) for MBC patients who have previously had trastuzumab-based therapy and are hormone receptor/HER2+) (Khan et al., 2020; Baselga et al., 2021). In phase III randomised trials was performed with lapatinib alone and lapatinib plus capecitabine in advanced MBC patients, capecitabine alone in a large phase III study of EGF100151 in HER2+ MBC patients undergoing trastuzumab-based therapy (median Z 4.4 months, hazard ratio [HR]). 8.4 months on average were deemed to be a lengthy period (Z 0.49 [95% CI: 0.34–0.71]; P 0.001). The median OS with capecitabine alone was 64.7 weeks (HR Z 0.87 [95% CI 0.70–1.08]; EA 0.206), while the median OS for lapatinib plus capecitabine was 75.0 weeks (Diéras et al., 2017; Baselga et al., 2021). This study demonstrated the long-lasting nature of the HER2 blockage caused by trastuzumab. In the EGF104900 research, patients with trastuzumab-refractory HER2+ MBC showed substantially longer progression-free survival (PFS) when compared to lapatinib alone (HR Z 0.74 [95% CI: 0.58–0.94]; PZ 0.011). When treated bilaterally, BC survival rates also improved (HR Z 0.74 [95% CI 0.57–0.97]; PZ 0.126) (Diéras et al., 2017; Baselga et al., 2021). The study also showed that the HER2 blockade induced by trastuzumab is sustainable. Progression-free survival (PFS) was significantly improved in patients with trastuzumab-refractory HER2+ MBC in the EGF104900 study (HR Z 0.74 (95% CI: 0.58–0.94); PZ 0.011) compared to lapatinib alone. BC survival rates also improved [HR Z 0.74 (95% CI 0.57–0.97); PZ 0.126] after bilateral therapy. Both lapatinib with capecitabine and trastuzumab with capecitabine were shown to have a significantly shorter median PFS (secondary endpoint) compared to trastuzumab + capecitabine in Phase 3 CEREBEL [RR 1.30 (95% CI: 1.04–1.64); *p* = 0.021] (Diéras et al., 2017; Baselga et al., 2021). Patients who took lapatinib plus capecitabine had a significantly shorter median progression-free survival (PFS) than those who got TDM1 in the historic Phase III EMILIA TDM1 study median Z 6.4 versus 9.6 months [HR Z 0.65 (95% CI: 0.55–0.77); *p* 0.001]. Prior to the second interim analysis’s consideration of crossings, TDM1 increased median overall survival by 5 months [HR Z 0.68 (95% CI: 0.55–0.85); *P* 0.001] (Diéras et al., 2017; Baselga et al., 2021). Overall survival (OS) was higher in the TDM1 group after crossover 29.9 months versus 25.9 months (HR Z 0.75 [95% CI: 0.64–0.88]; *p* 0.001). Table 4 than in the lapatinib control group, according to the study’s final descriptive analysis (Verma et al., 2012). In a phase 3 study, lapatinib in combination with AI improved progression-free survival (PFS) in patients with hormone receptor-positive/HER2+ MBC compared to AI alone. A better PFS was observed with lapatinib with trastuzumab with AI than with trastuzumab with AI in the alternative phase 3 study (Verma et al., 2012; Diéras et al., 2017; Baselga et al., 2021). In the original phase II single-arm study of lapatinib monotherapy, diarrhoea, nausea, and rash were the most common AEs (adverse events). According to a follow-up randomised trial, diarrhea, PPE (palmar–plantar erythrodysesthesia), nausea, rash, vomiting, and tiredness were the most common AEs of lapatinib in combination with capecitabine (Baselga et al., 2021). With capecitabine alone, PPE syndrome, nausea, diarrhoea, tiredness, vomiting, lack of appetite, and rash were the most common AEs.

### 3.2 Neratinib

Neratinib and lapatinib together were shown to be more efficient and well accepted than lapatinib alone in salvage therapy (Wang et al., 2021). 68 patients with HER2-positive MBC who had previously received trastuzumab and lapatinib were treated with neratinib and capecitabine (Chan, 2016). The patient’s ORR (overall recovery rate) was 57%, with a PFS of 35.6 weeks. The PFS in both was the same (nine months) (Dai et al., 2021). The ORR for the 19 participants in the study was 63%. The most common dose-limiting side effects (DLTs) were diarrhea and nausea. In the NALA study, neratinib capecitabine significantly outperformed lapatinib capecitabine in terms of PFS and time to intervention for CNS illnesses. For adult patients with metastatic HER2+ who have BC had two or more prior anti-HER2-based regimens in the metastatic setting, the FDA authorised neratinib (in combination with capecitabine) on 25 February 2020 (Dai et al., 2021). The NEfERT-T experiment was split into cohorts 3A (untreated lapatinib) and 3B to reduce the incidence of CNS cancer recurrence (treated lapatinib). The neratinib and trastuzumab were combined for the patients (Awada et al., 2016). 49% of cohort 3A and 33% of cohort 3B had a CNSORR composite of at least 50%. In terms of PFS and mOS, cohort 3A performed the best. The duration of cohorts 3A and 3B was 5.5 and 15.1 months, respectively. NALA results demonstrated that neratinib capecitabine were less effective than lapatinib capecitabine for the treatment of CNS disease (*p* = 0.043) (Dai et al., 2021).

The ExteNET, which began its trastuzumab-based full adjuvant therapy in early BC and is now eligible for random (1:1) treatment with neratinib or placebo, was open to more than 2,800 patients at 40 international institutions (Martin et al., 2017). The expense of Neratinib Group’s invasive disease-free survival (iDFS) over two and five years, respectively, was high (Martin et al., 2017). Neratinib, an oral next-generation TKI that permanently blocks HER1 and HER2, has shown encouraging antitumor results in individuals who had previously received it (Awada et al., 2016). It has an antiproliferative effect on cell cycle arrest because it is affixed to the receptor kinase’s ATP binding site *via* covalent coupling. PRB (phosphorylation of the retinoblastoma protein) and cyclin D1 phosphorylation levels were dropped. The neratinib has been approved by the FDA and EDQM (European Directorate for the Quality of Medicines) as extended adjuvant therapy for patients with early-stage breast cancer based on good 5-year statistics from ExteNET (Martin et al., 2017). Primary antitumor efficacy was observed in patients with breast cancer and CNS metastases who were given neratinib in combination therapy. Neratinib’s most common side effects were diarrhoea and nausea (Jacobs et al., 2019). Neoadjuvant therapy for HER2+ early-stage BC patients was studied in NSABP FB7 (NCT01008150) (Jacobs et al., 2019). The paclitaxel, doxorubicin, and cyclophosphamide were administered every week along with trastuzumab and/or neratinib. Trastuzumab (38%) and neratinib (50%) each had a higher cancer diagnosis rate, but combination therapy had a 50% higher rate (33%). In the ISPY2 (Investigation of serial studies to predict your therapeutic response with imaging and molecular analysis2) research, neratinib was found to be effective in identifying tumour subgroups that were responsive to the treatment. The combination of neratinib and capecitabine was investigated in a phase I/II study. There was an ORR of 64% (95% CI, 51–76%), and stable disease progression was observed in 8% of the patients. The patients who had previously received lapatinib experienced an ORR of 57% (95% CI, 18–90%), with one patient achieving a CR, and stable disease was seen in 14%. The median PFS in patients who had not received prior lapatinib was 40.3 weeks (95% CI, 30.6–36.0) and 35.9 weeks (95% CI, 18.9–60.1), respectively. A third phase III trial is currently ongoing to confirm the efficacy of neoadjuvant therapy (Chan, 2016; Jacobs et al., 2019). This study was no longer considered a Phase 3 study when the trial’s number of participants decreased from 1,200 to only 480 participants. In this trial, women with metastatic HER2+ BC who had not previously been treated received neratinib, trastuzumab, and paclitaxel (Awada et al., 2016). The median PFS for both groups was 12.9 months. These two groups had similar outcomes in terms of ORR (clinical benefit rate, and DOR (duration of response) (Awada et al., 2016). Lapatinib plus capecitabine was compared to neratinib plus capecitabine in patients with metastatic HER2-positive BC, who had received at least two prior lines of therapy as part of the Phase 3 NALA trial in patients with metastatic HER2+ BC who had received at least two prior lines of therapy (Dai et al., 2021). Neratinib patients had a better prognosis (hazard ratio 0.76, 95% CI = 0.63–0.93, *p* = 0.0059) than those in the placebo group. It was found that patients with an HR infirmity benefited most from this combination (HR hazard ratio: 0.76, 95% confidence interval: 0.57–1.01; HR + hazard ratio: 0.94, 95% confidence interval: 0.72–21). In this TKI study, patients with HR + illnesses had a better DFS HR + hazard ratio of 0.51 vs. 0.93 than in the extended study. After two anti-HER2 treatments, the FDA approved the combination of neratinib and capecitabine (Awada et al., 2016; Dai et al., 2021).

### 3.3 Pyrotinib

An irreversible dual pan-ErbB receptor tyrosine kinase inhibitor called pyrotinib was created to treat advanced solid cancers that were HER2-positive. This TKI can be used with capecitabine, a drug approved for the treatment of advanced or metastatic HER2+ breast cancer in China in 2018 (Wang et al., 2022). Lapatinib and capecitabine were contrasted with pyrotinib and capecitabine in phase 2 research, with response rates of 78% and 57%, respectively. The median PFS for the pyrotinib and lapatinib groups was 18 and 7 months, respectively (Diéras et al., 2017; Wang et al., 2021). The effects of pyrotinib and capecitabine were compared to the effects of pyrotinib and capecitabine, as well as a placebo and capecitabine, after the third phase of the study (Li et al., 2021; Wang et al., 2022). The pyrotinib group had a median PFS of 11 months, while the placebo group had a median PFS of 4.1 months. The patients were then treated with pyrotinib monotherapy and had a singl-agent response rate of 38% with a median PFS of 5.5 months (Li et al., 2021; Wang et al., 2022). The median PFS for pyrotinib was 12.5 months, while that of lapatinib was only 6.5 months (6.8 months, *p* = 0.0001) (Li et al., 2021; Wang et al., 2022). Patients were more likely to experience diarrhoea and hand–foot syndrome. Several studies are now being conducted on the potential use of pyrotinib, which has not been tested in other countries (Li et al., 2021; Wang et al., 2022). Pyrotinib monotherapy was first tested in TKI-naive HER2-MBC patients in Phase I dosage escalation studies (Li et al., 2021; Wang et al., 2022). Only half of the participants in this study had a one-to-one odds ratio, relapsed or metastatic cancer patients who had previously been treated with taxanes and anthracyclines were randomly assigned to receive pyrotinib with capecitabine (Li et al., 2021; Wang et al., 2022). Trastuzumab was given to more than half of the patients as an adjuvant or metastatic treatment. Pertuzumab, or TDM1, was completely omitted from the treatment regimens (Liao et al., 2021). Two of the four medications studied had an ORR of 79%, with a median PFS of 18.1 months for the two therapies and 7.7 months for the other two; the other two had a PFS of 7.7 months (Liao et al., 2021). China is now conducting a phase III clinical trial for HER2 MBC, which is currently being tested with pyrotinib (Liao et al., 2021).

### 3.4 Afatinib

Afatinib is a pan-HER TKI that is irreversible and has a strong affinity for EGFR, A phase III trial using afatinib to treat HER2 was proposed to early stop recruiting by an independent data tracking group (Hurvitz et al., 2014; Wind et al., 2017). In the initial analysis of open-label, phase III LUX-Breast 1 trial the median PFS for the afatinib and trastuzumab groups was 5 months and 6 months, respectively (HR Z 1.10; 95% CI: 0.86–1.41; PZ 0.43) (Hurvitz et al., 2014; Wind et al., 2017; Hickish et al., 2022). Five percent of afatinib patients and 3% of trastuzumab patients had dosage decreases because of AEs. Treatment was required for 15% of patients in the afatinib arm and 7% of participants in the trastuzumab arm (Hurvitz et al., 2014; Wind et al., 2017; Hickish et al., 2022). One-quarter of patients in the afatinib group had their dose reduced because of diarrhea, rash, nausea, tiredness, and stomatitis were the most common AEs of any grade. As compared to the trastuzumab group, the afatinib group saw greater incidences of PPE syndrome (12% as opposed to 1%). Diarrhea, rash, fatigue, stomatitis, mucosal infection, and hypokalemia were the most commonly reported grade III–IV adverse events (AEs Hurvitz et al., 2014; Wind et al., 2017; Hickish et al., 2022).

## 4 TKIs and Brain Metastasis

Treatment for brain metastases is challenging because of its heterogeneity (Darlix, et al., 2019; Kuksis et al., 2021). According to reports, following therapy with ado-trastuzumab emtansine, at least a 30% reduction in the size of CNS lesions was seen in 42.9% of patients with identifiable brain metastases (*n* = 126) in the KAMILLA trial (Montemurro et al., 2020; Kuksis et al., 2021). Patients who did not have radiation therapy for their brain metastases observed a decrease in the size of their tumours of at least 30% in 49.3% of the patients. TKIs are used as a promising treatment choice for HER2+ BC brain metastases (Nader-Marta et al., 2022). In clinical research involving 242 patients with HER+ BC brain metastases, 20% of those receiving capecitabine and lapatinib experienced CNS ORR. In Section 2 of the LANDSCAPE trial, which comprised 45 patients, 65.9% of those with untreated BC brain metastases showed a partial intracranial response (Montemurro et al., 2020; Kuksis et al., 2021). The progression-free survival rates for patients with BC brain metastases in both trial arms were equivalent for those who received capecitabine with lapatinib vs adotrastuzumab emtansine, according to the EMILIA research’s retrospective analysis (Montemurro et al., 2020; Kuksis et al., 2021). In contrast, capecitabine and lapatinib were found to have a PFS of only 6 vs. 4 months (risk ratio 0.65; *p* = 0.001). Patients with CNS illness who received adotrastuzumab emtansine saw a significant improvement in OS (26.8 vs. 12.9 months, risk ratio of 0.38, *p* = 0.008). 540 patients with metastatic HER2+ breast cancer received either capecitabine plus lapatinib or capecitabine plus trastuzumab in the third CEREBEL study (Patel et al., 2020; Tan et al., 2021). For patients receiving lapatinib and trastuzumab, the onset of brain metastases was the first sign of relapse in 3% and 5% of patients, respectively (*p* = 0.360). Patients receiving trastuzumab had more serious adverse events and a longer PFS and OS than those receiving lapatinib (Li et al., 2020). Neratinib has also been tested in patients with brain metastases. In 49 patients with BC brain metastases who had previously received lapatinib and were treated with neratinib and capecitabine in a Phase 2 study, the CNS ORR was 49%, compared to 33% for those who had taken the medicine before (Dai et al., 2021). In the research, NEfERT-T (neratinib + paclitaxel vs. trastuzumab and paclitaxel), 8.3% of patients in the neratinib group and 17.3% of patients in the trastuzumab group had symptomatic or progressive CNS disease with cumulative brain metastases (Awada et al., 2016; Dai et al., 2021). Neratinib patients were 20% more likely to be diagnosed with cancer than trastuzumab patients (*p* = 0.002). There were twice as many CNS involvements in the trastuzumab group as there were in the neratinib group. These individuals had already been diagnosed with symptoms before the study, since NrfERTT did not involve a test for brain metastases at baseline 50. The third phase of the NALA research comprised patients with stable or asymptomatic brain metastases (Awada et al., 2016; Dai et al., 2021). Neratinib capecitabine had a shorter PFS and CNS intervention time than lapatinib capecitabine in study participants with stable, asymptomatic brain metastases. In the NALA study (*p* = 0.043), a CNS intervention was necessary for 22.8% of patients treated with neratinib and 29.2% of individuals treated with lapatinib. For patients with HER2-positive brain metastases, these trials suggest that neratinib may be more effective than lapatinib in accessing the central nervous system (CNS) and in treating the metastases. (Awada et al., 2016; Dai et al., 2021). The combination of tucacitabine, trastuzumab, and capecitabine was tried in the HER2 CLiMB (Clinical Trial Multi-analyte Blood Test) trial and demonstrated exceptional outcomes. This trial was more precise than earlier ones since it included 291 patients with active or strong brain metastases (198 in the Tucatinib group and 93 in the placebo group). 22% of patients had untreated metastases, while 37% of patients had dealt with and advanced from a CNS illness. Tucatinib patients had a 68% lower risk of intracranial growth or death. The intracranial ORR between the tucatinib group and the modification group differed significantly (*p* = 0.03) (Lin et al., 2020). The indication statement for tutatinib is the first for an FDA-approved medication to include patients with brain metastases. These results have increased interest in tucatinib as a first-line metastatic therapy for individuals with BC brain metastases (Lin et al., 2020). The TOPAZ study (NCT04512261) is evaluating the combination of tucatinib with pembrolizumab and trastuzumab in patients with HER2+ BC brain metastases (Garcia-Alvarez et al., 2021).

## 5 Mechanisms of resistance to HER2-directed therapies

In the past 2 decades, TKIs and ADCs (antibody-drug conjugates) have been used in chronic-stage patients with HER2+ MBC and have shown improved clinical outcomes; however, HER2+ MBC is still a difficult case to treat (Koster et al., 2022). In recent years, resistance to anti-HER2 therapies has been observed in genetic heterogeneity, reprogramming of activated intracellular signal proteins, modulation of immune regulation, metabolic dysregulation, constitutive activation of HER2/HER3/HER4, reactivation of PI3K/AKT/mTOR (serine/threonine protein kinase in the PI3K-related kinase), and modulations in drug binding to HER2 (Ruiz-Saenz et al., 2018). Lapatinib, as well as lapatinib in combination with trastuzumab and T-DM1, have been proven to be ineffective against the HER2 L755S mutation (Sung et al., 2018; Liao et al., 2021). TKI resistance is brought on by HER2-L755S mutations because of increased MAPK and PI3K/AKT/mTOR pathway activity (Li et al., 2019). Downregulated expression of poly (rC)-binding protein (PCBP1) has been observed in various types of cancer patients. The p27 protein binds to PCBP1 at the 3′-UTR of p27mRNA and stabilizes PCBP1 at the 3′-UTR of p27mRNA (Shi et al., 2018). Reduction in p27 expression at the transcriptional level causes loss of PCBP1, which may lead to lapatinib resistance in BC cells. The tumorigenesis process and persistence of HER2+ BC cells are prevented by adverse reactions to IGF2/IGF-1R/IRS1, and anti-IGF-1R combination therapy is resistant to trastuzumab (Wu et al., 2022). Dihydromyricetin induces expression of miR-98-5p and reduces the expression level of IGF2, which may reverse the resistance of HER2+ BC cells to trastuzumab (Zhang et al., 2022). Clinical management of HER2+MBC requires personalised and accurate research on drug resistance mechanisms for monotherapy and combination therapy (Luque-Bolivar et al., 2020), this can provide new insight for novel drug development and clinical applications, Resistance to monoclonal antibodies is caused by HER2 expression, high levels of p95HER2, and PTEN loss (Luque-Bolivar et al., 2020; Wu et al., 2022). The S310F mutation of HER2 leads to the serine substitution at amino acid 310 with phenylalanine, resulting in pertuzumab resistance (Zhang et al., 2019). The average length of stay (DOR) was 8.4. There was also an 8.3-month PFS median in this study (Zhang et al., 2019). In this context, several TKIs are being studied. Resistance to medicines targeting HER2 has been explained in terms of both innate and acquired mechanisms (Rexer and Arteaga, 2012). Resistance to lapatinib can be acquired through the L755S pathway, and *in vitro* evidence of cross-resistance to patinib has been provided. Some cells appear to be resistant to both neratinib and panHER2TKI (Breslin et al., 2017). The clinical importance of these mutations has to be studied further so that treatment can be tailored to individual variants. A further mechanism for resistance is the overexpression of some HER family receptors when HER2 family receptors are partially blocked (Gaibar et al., 2020). Adding pertuzumab to trastuzumab or combining it with a powerful TKI focused on targeting multiple receptors in the HER2 family can help overcome this mechanism (Ishii et al., 2019). AXL (AXL receptor tyrosine kinase) activation is responsible for mediating this pathway. It is possible to circumvent this resistance mechanism *in vitro* with the use of multikinase inhibitors such as foretinib, an AXL inhibitor (Goyette and Cote, 2022). Resistance mechanisms can also be defined as changes in the signalling pathways that follow. Tumor growth can be triggered by activating mutations in PIK3CA (Phosphatidylinositol-4,5-Bisphosphate 3-Kinase Catalytic Subunit Alpha) and poor expression of tumour suppressor genes (such as PTEN) (Fusco et al., 2021). Resistance to HER2-targeting medicines can also be attributed to the cyclin pathway. Targeting cyclin-dependent kinases 4 and 6 in the preclinical stage has been shown to restore sensitivity to HER2-targeted treatments (O’Brien et al., 2020). Another recognised mechanism of resistance to cancers that express both HER2 and ER is the bidirectional interaction between the two receptors (Rani et al., 2019). *In vitro*, blocking both HER2 and estrogen receptors can prevent this phenomenon from occurring (Rani et al., 2019; O’Brien et al., 2020).

## 6 Nanotechnology to treat HER2-positive BC

**FIGURE 6:**
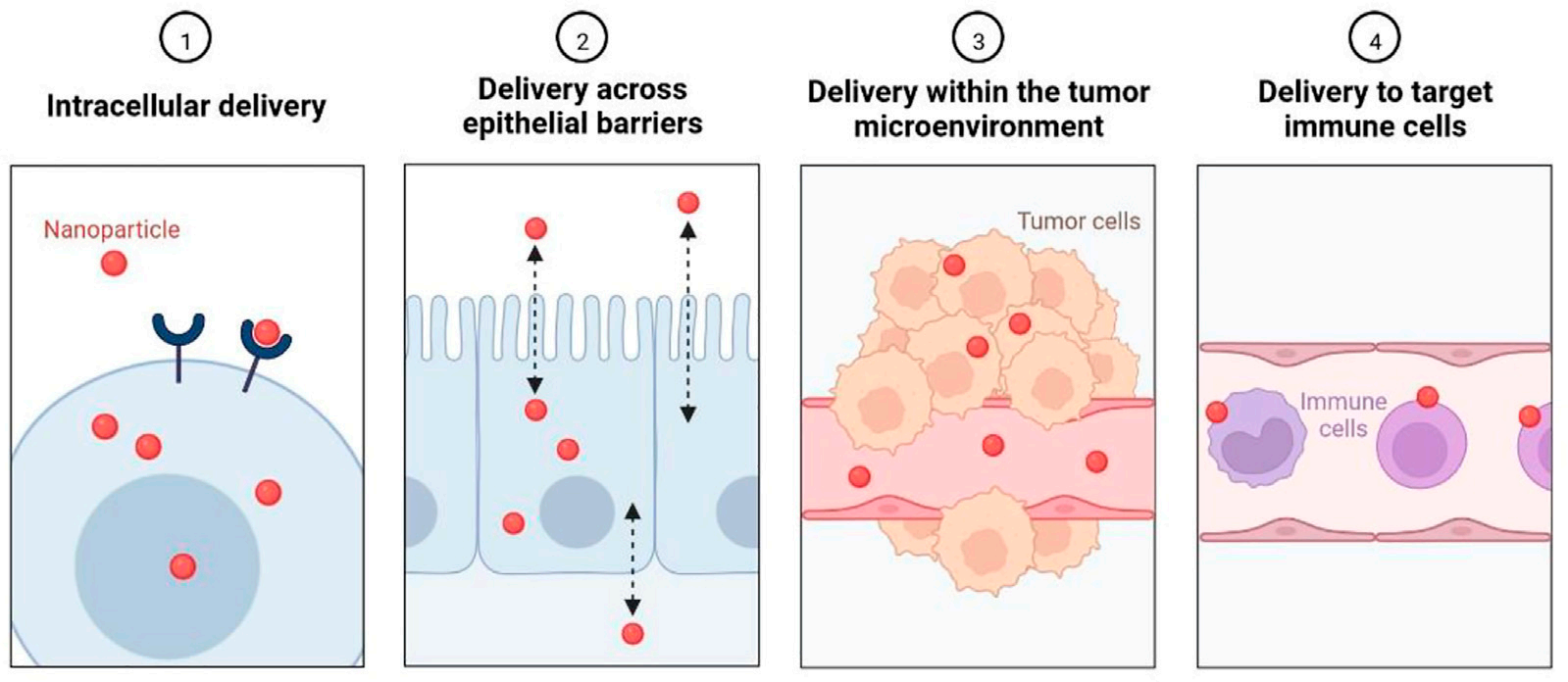
Biological barriers that nanoparticles can help overcome.

**FIGURE 7:**
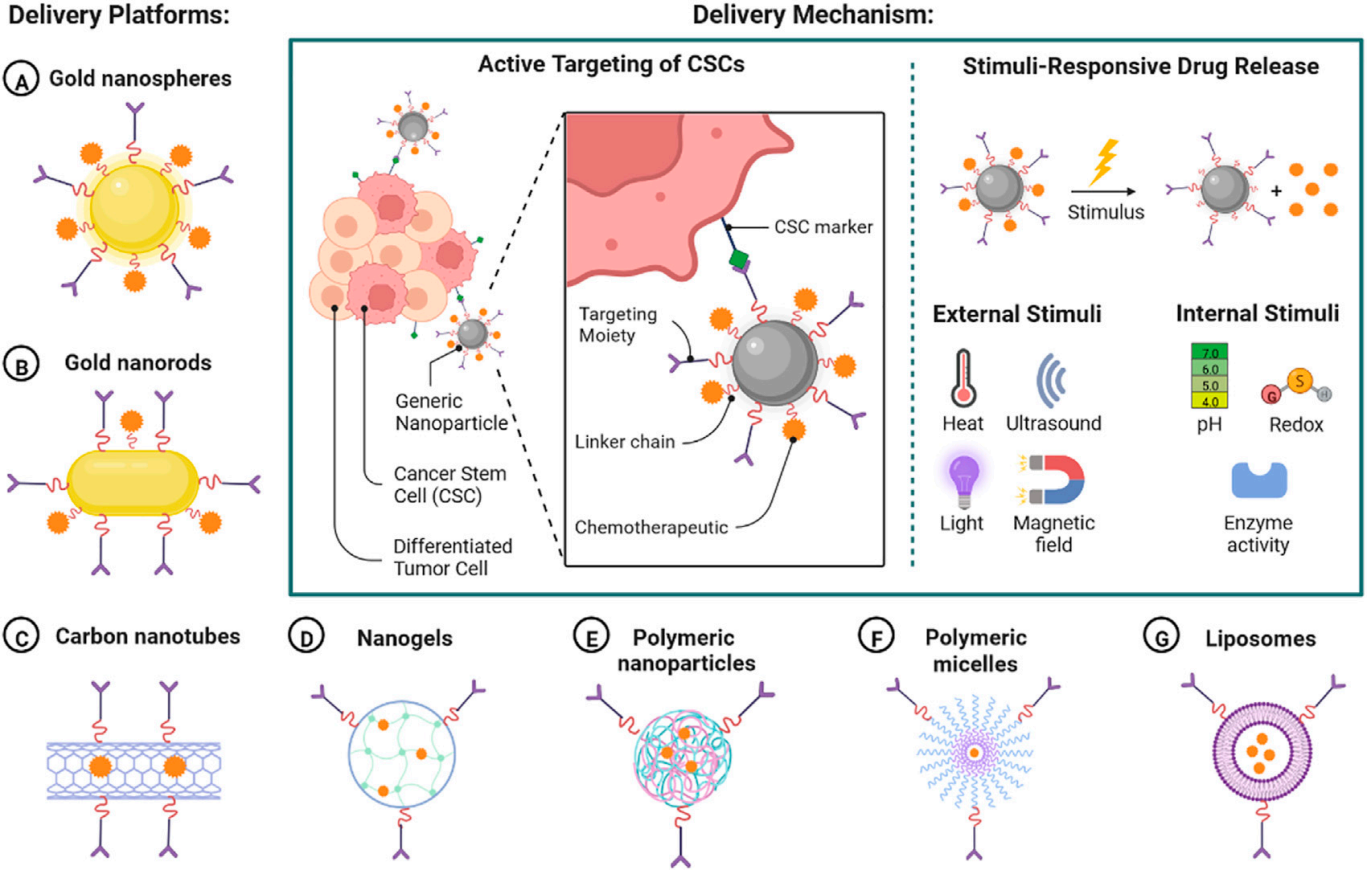
Nanoparticle-mediated targeted drug delivery to cancer stem cells (CSCs).

Current treatment option has shown limited efficacy in patient with HER2+ BC and causes adverse rection and rapid drug resistance, so the urgent requirement is targeted treatment for effective clinical outcome (Yang et al., 2022). Nanoscience and technology could have important role in early detection, targeted drug delivery to reduce disease burden. In the current scenario, to overcome the limitations of HER2+ BC therapy, various nano-based safe and targeted therapeutic agents are being developed (Cheng et al., 2021). Nano formulation-based targeted therapy can be developed by intracellular delivery, delivery across the epithelial barrier, delivery with the tumour microenvironment, and delivery to targeted immune cells, as shown in Figure 6 (Yu et al., 2022; Yao et al., 2020). There are various types of delivery platforms used to deliver nano formulation including gold nanoparticle, gold nanorods, Corban nanotubes, nanogel, polymeric nanoparticles, polymeric micelles, and liposomes (Tang et al., 2021). Nano formulations are delivered by a mechanism by targeting specific markers (Patra et al., 2018). The cancer stem cells (CSC) have differentiation and self-renewal capability and act as tumour inducing agent. It can be also used as a potent target for anticancer agent with nanomedicine in patent with HER2+BC (Wu et al., 2017). Nanoparticle-Mediated Targeted Drug Delivery to Cancer Stem Cells (CSCs) are shown in Figure 7.

Newly identified immune-based treatment options that regulate the host immune response to target the cancer cell and remove metastatic tumour (Gun et al., 2019) This immunotherapeutic based approach is based on chimeric antigen receptor T-cells (CAR-T), cytokines, immune checkpoint inhibitors (ICIs) and cytokines (Pan et al., 2022). The ICIs are delivered to single check point inhibitors to AEs and drug resistance (Bagchi et al., 2021). Anti-CTLA4 (anti-PD1) treatments are used to promote peripheral T cells which strengthen the immune system. Poly (-L-Malic Acid) (PMLA) based polymeric scaffolds are used to deliver anti-PD1 immunoconjugates, which increased the survival rate in mice (Galstyan et al., 2019). Nano formulations can be used to reduce drug dose concentrations. Poly (Lactide-O-Glycolic) Acid (PLGA) conjugated n with PEG NPs and are also used to deliver anti-Transforming Growth Factor-Receptor 1 (TGF-R1) to reduce the activity of TGF-β, which reduces the tumour growth and increased the survival rate (Schmid et al., 2017). Anti-CTLA-4 conjugated with Iron-Oxide Nanoparticles (IONPs) reduce the tumour burden in 4T1 mice. 4T1 mice are mixed with tumour cells in infiltrates of metastatic organs. Nano-formation based therapeutic approach is more effective including radiotherapy, photothermal therapy and photodynamic therapy, which can reduce the tumour burden (Chen et al., 2020). There are various effective nanoparticle conjugated plant extracts that have been identified with less toxicity (Dhupal and Chowdhury, 2020). Nanomaterials-based drugs are effectively delivered to site of target and provided effective control of malignant tumours (Sadat et al., 2015). The use of anti-HER2 ligand in various nano formulations to target HER2 receptors (Table 5). It is about to begin Phyto molecules are easily defused through the cell membrane, intracellular organs and induce oxidative stress. Nanoparticles-based Phyto molecules have transformed the HRE2+ BC therapy significantly improving clinical outcome (Jiang et al., 2021). All these approaches have shown significant outcomes and reduced the tumour microenvironment burden.

**TABLE 5:** The use of anti-HER2 ligand in various nano formulations to target HER2 receptors.

Nanocarrier	Therapeutic agent(s)	Conjugates	Clinical outcome	References
HER2 immunoliposomes and liposomes in combination	Bevacizumab in a liposome and doxorubicin in an immunoliposome	Inhibition in HER2/MDR BC patiemts	Reduced the tumour size and lower toxicity	Tang et al. (2021)
Polymalic acid based nano drug	Antisense oligonucleotides	The polymer-attached 12-mer peptide mimicking trastuzumab recognises HER2+ cells	Decrease the tumour size	Ding et al. (2017)
Ethylenediamine functionalized single-walled nanotube	Oncogene suppressor p53	Increased uptake by MCF-7 cells	Leading to enhanced caspase-3-induced apoptosis	Karmakar et al. (2011)
HER2 antibody-coated gold nanoparticles and gold sulphide	Gold–gold sulphide for high- intensity photoablation	Bind with SK-BR-3 cells overexpressing HER2	Promotes thermal damage to tumour	Day et al. (2010)
Trastuzumab-modified gold nanoparticles with 111-In labelling	Radioactive-111-in	Local its injection to mice with sc MDA-MB-361	Tumours arrested	Chinen et al. (2015)

## Conclusion

HER2+ BC patients are treated with TKIs, but many questions remain unanswered, particularly in terms of the drug combinations’ efficacy and safety, as well as their side effects and toxicities. Another unresolved subject is how to select a TKI for anti-HER2 therapy based on prior treatment. TKI’s efficacy and distinct toxicity profile will be determined in ongoing studies, as well as the function it plays in treating HER+ breast cancer. In addition, the precise role of TKIs in the escalation of treatment is still a mystery. There is still a problem with the treatment plan. First-line medications may have a better response if administered in this manner. The optimal quality of life for the patient population can only be achieved by answering the numerous questions that remain unanswered.
